# Practical Notes on Diagnosis and Treatment

**Published:** 1906-12-29

**Authors:** 


### PRACTICAL NOTES ON DIAGNOSIS AND TREATMENT.

**Pilocarpine in Hiccough.**

HYDROCHLORIDE of pilocarpine dissolved in water is said to be useful in cases of nervous and hysterical hiccough. Ten minims of a 1 per cent. solution may be given by the mouth every three or four hours.

**Vomiting of Pregnancy.**

THE following is recommended in the vomiting of pregnancy—namely, menthol 1 grain, rectified spirit 30 minims—dissolve and add syrup of orange 30 minims. Take a teaspoonful every two or three hours.

**Morphine in Kidney Disease.**

WE are taught to be afraid of giving morphine in renal disease, nor do I wish to induce you to be incautious, but there is no doubt the danger is much exaggerated; you may use opium with care, but should be careful of hypodermic injections.—*Dr. Goodhart.*

**Rupture of the Perinæum.**

MANY plans for preventing rupture of the perinæum as the head passes through the vaginal orifice are given, but the best plan of all is to let it alone. The only help that seems to be of any real use is when between the pains it is possible to slip the perinæum back over the forehead.—*Dr. Peter Horrocks.*

**On Rectal Examination.**

BEFORE making a digital examination of the rectum notice if the buttocks are drawn tightly together, needing some force to separate them; also notice if the anus is tightly contracted up. If these two conditions are present they are more than suggestive of fissure. On the other hand, in carcinoma of the rectum the anus is found to be patulous.—*Sir Frederick Treves.*

**Corneal Ulcers.**

IN the small grey central ulcers of the cornea accompanied by much photophobia and seen in children weak mercurial ointment is most useful. Mr. Hutchinson considers it a specific.

**Herpes in Pneumonia.**

HERPES is often absent in pneumonia the result of septic causes, and even also in cases dependent on other causes. As a matter of clinical experience I prefer to see herpes present from the prognostic point of view.—*Sir Dyce Duckworth.*

**Spasmodic Dyspnœa in Mitral Disease.**

NITRITE of amyl or nitroglycerine give some relief at times, but they are far inferior to atropine. This is of great service, but it must be given in sufficiently large doses—say a hypodermic injection of $\frac{1}{25}$ grain. When this fails venesection or leeches may give relief.—*Dr. Lees.*

**Lotion for General Eczema.**

IN general eczema a useful method of treatment is to order a lotion consisting of $\frac{1}{2}$ oz. of solution of subacetate of lead and $2\frac{1}{2}$ oz. of liquor carbonis detergens. A teaspoonful of this should be added to a pint of tepid water, which is then used to sponge the whole of the body.—*Sir Stephen Mackenzie.*

**Foreign Body in the Ear.**

WHEN you are dealing with a child in whose external auditory meatus there is a hard foreign body, you should resort to no treatment beyond syringing. A hard foreign body in such a position is harmless when left alone, and as the child grows and the external meatus gets larger the day will surely come when the foreign body can be removed by gentle syringing.—*Mr. Marmaduke Shield.*

